# Effects of mesh surgery on sexual function in pelvic prolapse and urinary incontinence

**DOI:** 10.1590/S1677-5538.IBJU.2019.0618

**Published:** 2020-11-18

**Authors:** Gökmen Sukgen, Adem Altunkol, Ayşe Yiğit

**Affiliations:** 1 Sukgen Gynecology and Obstetrics Clinic Adana Turkey Sukgen Gynecology and Obstetrics Clinic, Adana, Turkey; 2 University of Health Sciences Adana City Teaching and Research Hospital Department of Urology Adana Turkey Department of Urology, University of Health Sciences, Adana City Teaching and Research Hospital, Adana, Turkey; 3 Special Megapol Hospital Gynecology and Obstetrics Clinic Istanbul Turkey Special Megapol Hospital, Gynecology and Obstetrics Clinic, Istanbul, Turkey

**Keywords:** Pelvic Organ Prolapse, Urinary Incontinence, Sexual Behavior

## Abstract

**Purpose:**

We aimed to determine pre-operative and post-operative sexual function scores of patients who underwent four-arm polypropylene mesh implantation surgery to treat urinary incontinence and pelvic organ prolapse.

**Materials and Methods:**

A prospective study from January 2011 to November 2015 including patients (n: 72) submitted to surgical mesh implantation (four-arm anterior mesh implant (Betamix POP4®, Betatech Medical, Turkey) questioned the patients with Female Sexual Function Index evaluation form. The questionnaire was applied to all patients at pre-operative, post-operative 3rd month and post-operative 1st year periods.

**Results:**

The mean age of the patients was 47.2±7.1 years. The mean Body Mass Index (kg/m2) was 28.7±3.7. The average of incontinence duration (year) was 4.6±2.6 and the average for operation time (min) was 35.7±2.1. After the urinary incontinence and pelvic organ prolapse surgery, it was observed that incontinence complaints of patients reduced. Furthermore, there was a positive change in quality of life and sexual function of patients at the post-operative period. There was a statistically significant increase according to Female Sexual Function Index score among all three periods (16%, 86% and 100% respectively, p=0.001) and improvement of sexual functions was observed.

**Conclusions:**

Transvaginal mesh use in the surgical treatment of pelvic organ prolapse improves quality of life. However, risk factors such as transvaginal mesh usage indication, surgical technique and experience of the surgeon, suitability of the material, the current health status of the patient and postoperative personal care of the patient may affect the success of operations.

## INTRODUCTION

Sexuality is a complex process associated with neurological, vascular and endocrine systems (1). Sexuality is one of the main factors affecting the general health status and quality of life of women (2, 3). Sexual dysfunction in women is a complex problem that is affected by many biological, psychological and individual factors. Sexual dysfunction in women is a common problem that increases with age and affects approximately 30% to 50% of women. However, the prevalence among young women is quite high.

Pelvic organ prolapse (POP) is a type of pelvic floor disorder seen in about one-third of all women. Pelvic organ prolapse refers to the outflow or sagging of the bladder, uterus, vagina, small intestine, or rectum of the pelvic floor organs, down the vaginal canal or anus as a result of prolapse. Pelvic organ prolapse, usually in postmenopausal or climacteric period, may be observed sometimes in young women who wish to maintain fertility (1–4). Pelvic floor disorders such as urinary incontinence/pelvic organ prolapse are seen commonly among women at all ages and are not just a long-lasting medical problem but also has negative physiologic, psychological, hygienic, economic and sexual effects which all can be summed up under the term “quality of life” (5). POP incidence is increased with age and POP surgery rates show an increase with time. The aim of POP surgery is to correct the anatomy, symptoms and functions. It is notable that POP surgery increases quality of life (2). POP and urinary incontinence (UI) are common complaints and may present together in the same patient. The most common type of UI is urge - incontinence. SUI is the most frequent type of UI only among middle-aged women which is observed at high intraabdominal pressure conditions such as effort, exertion or sneezing or coughing and defined by International Continence Society (ICS) as urinary incontinence, observed when intravesical pressure exceeds urethra pressure with no increase in detrusor muscle activity (6). It was observed that 63% of women having SUI have also a descensus while 62% of women having a descensus have SUI at the same time (7); also, urinary incontinence can be observed in the first three years of menopause at 10% of women having no complaints previously (8). Vaginal mesh application surgeries came up at POP treatment in addition to conventional pelvic reconstruction methods (9). The first vaginal use of mesh was done by Julian et al. in 1996 and they reported significantly fewer complications and recurrence rates (10). In our country, with increasing industrial pressure in the 2000s, there has been increasing mesh complications in mesh surgery with the contribution of patient incompatible mesh material such as monophilized micropore and insufficient surgical experience. In this context, the warnings of the FDA limiting the use of mesh were unavoidable. However, repairing the fascial defect at the base of the pelvic prolapse with its own fascia, as in conventional surgeries with a high recurrence rate, unfortunately leads to the need for a second surgery due to recurrence. Following studies also supported the place and importance of mesh in the treatment of POP (11, 12). There are variations at the results of pelvic floor surgery's effects on sexual function in literature. Quality of life may improve, stay the same or may become worse after vaginal surgery (13). In our country (Turkey) there are very few studies evaluating the UI and/or POP surgerie's effects on quality of life and sexual functions, which remained under-researched.

In our study, we aimed to compare preoperative and postoperative quality of life and sexual functions of patients having POP and SUI complaints and went through four-arm polypropylene mesh implantation surgery which is accepted as a new method in our clinic.

## MATERIALS AND METHODS

Local ethics committee (2018.01.31-11/155) approved this study and informed consent was obtained from all individual participants included in this study. A prospective study from January 2011 to November 2015 including patients in which type 1 polypropylene macropore surgical mesh (Betamix POP4®, Betatech Medical, Turkey) implantation were applied with indication of POP and UI and questioned with Female Sexual Function Index (FSFI) evaluation form at outpatient clinic controls with also some information gathered from their computer and file records. Anamneses were taken and routine physical examination, urine culture and office cystometry were done for all patients. This study was conducted at a single center. All operations were performed by a single surgeon (GS).

All urodynamics were performed using 7F transurethral and rectal balloon catheters according to the International Continence Society standards (14). Patients were standardly asked to empty their bladder before this study, and the first step was to measure the postvoid residual (PVR) volume taken from the 7F urethral catheter placed. Filling cystometry was performed with saline. Decreased rate of 30mL/min in cases of severe detrusor overactivity (DO) or known small functional capacity were used in most of the patients included in the present series. A pressure-flow study was obtained at selected times as determined by the clinical scenario (i.e., filling, Valsalva, void/attempt to void, after voiding). PVR was reassessed after the pressure-flow study. A blind review of all the urodynamic results was done by a urodynamic expert. The following urodynamics data, defined according to the International Continence Society guidelines (14) were collected: cystometric capacity (mL), volume at first sensation of bladder filling (mL), volume at first uninhibited detrusor contraction (mL), the pattern of DO (phasic vs. terminal), maximum flow rate (Qmax, mL/sec), detrusor pressure at maximum flow (PdetQmax, cm H2O).

The FSFI scoring form was applied to all patients at pre-operation, post-operative 3rd month and post-operative 1st year periods. FSFI was developed by Rosen et al. (15) in the year 2000 and is a test of 19 questions about arousal, sexual desire, orgasm, lubrication, sexual satisfaction and pain. In addition, risk factors that may affect the sociodemographic, obstetric and sexual functions of women are questioned in the questionnaire. Age, age of marriage, education, occupation, education of the spouse, body mass index (BMI) were evaluated as demographic characteristics. The number of children, the type of the last birth, the method of family planning and the frequency of sexual intercourse (weeks) were questioned as obstetric features. Turkish validity and reliability test were carried out for the form by the Turkish Andrology Society in 2003 (16). The scoring is performed by multiplying each question's point with a given ratio and each part is evaluated out of 6 points with a minimum of 2 and a maximum of 36 points in total at the end (15, 16). Patients at different POP grades were included. Also, patients with POP had a clinic and cystometric diagnosed SUI. SUI was diagnosed by cystometry and POP grading was done by POP-Q classification clinically. According to POP-Q classification it was observed: Stage 0: No prolapse demonstrated, Stage 1: The most distal portion of the prolapse is greater than 1cm above the level of the hymen; Stage 2: The most distal portion of the prolapse is less than 1cm above or below the level of the hymen; Stage 3: The most distal portion of the prolapse is greater than 1cm below the level of the hymen, but protrudes no further than 2cm less than the total vaginal length Stage 4: Complete eversion of the total length of the lower genital tract The distal portion of the prolapse protrudes to at least 2cm less than the total vaginal length (17).

## SURGERY TECHNIQUE

Anterior four-arm mesh implant

Surgery was performed under general anesthesia with the patient in the lithotomy position with an indwelling urinary catheter. A linear incision was performed on the anterior vaginal mucosa, about 2.5cm below the external urethral opening, and space was dissected until reaching the bladder base. Vesicovaginal ligaments were retracted laterally. The proximal part of the mesh was passed over arcus tendineus fascia pelvis (ATFP) with an obturator fossa guide. The posterior part of the four-arm mesh was passed through the obturator foramen. The anterior arms were placed as a mid-urethral sling as in the intravaginal slingplasty (IVS) procedure, supporting the mid-urethra and bladder. Posterior fringes of the mesh were fixed on cardinal ligaments. About an 8-10cm piece of monofilament, inelastic polypropylene tape is attached to the underside of the vaginal apex. Polypropylene sutures are placed into both of the cardinal ligaments and threaded through the lateral edges of the apical sling and tied down, restoring apical support. Posterior mesh arms were fixed at the skin level by performing traction, but the anterior part was not fixed at the mid-urethra and skin level, and tension-free placement was performed.

Prophylactic preoperative antibiotics (cefazolin 1g, amoxicillin and clavulanic acid 1.2g or gentamycin 160mg) were administered intravenously. A urinary catheter was removed on the morning of the postoperative day. A vaginal gauze pack (gauze soaked in Betadine iodine) was placed for 12h. The post-voided residual urine was measured by ultrasonography before each patient was discharged. All the patients received topical intravaginal oestrogen cream treatment for at least twelve months following the operation (Ovestin® 1mg/gram daily).

## Statistical analysis

Patient's pre-operative, post-operative 3rd month and post-operative 1^st^-year results were compared and analyzed. All data were collected by detailed face to face questioning of patient's sociodemographic characteristics, medical and sexual history and risk factors which may cause sexual function disorders. Data were presented as the mean±standard deviation. In comparing results, paired student t-test was conducted to compare continuous variables, and chi-square test was conducted to compare categorical variables. The data were analyzed using “SPSS 21 for Windows” software and statistically significance was described by p value <0.05.

## RESULTS

Patients with stage 3 and 4 were included in the POP-Q classification. 91 patients were evaluated in total. Patients having a history of pelvic reconstructive surgery (n: 4), not treated infection (n: 3), urinary tract and genital organ lesions (n: 5), any pelvic cancer and/or a history of radiotherapy to the pelvic region (n: 7) were excluded from this study. This study included 72 patients with sexual dysfunction, POP and SUI complaints. The mean of patients’ age was 47.2±7.1, BMI (kg/m2) was 28.7±3.7, incontinence duration (year) was 4.6±2.6, operation time (min) was 35.7±2.1 (Table-1). According to the distribution of the POP degree, 48 patients had anterior wall, 12 patients had anterior+apical wall and 12 patients had anterior+posterior wall defect.

**Table 1:** Demographic characteristics of patients.

Demographic characteristics		(n: 72)
Age (year) (Mean+std deviation)		47.2±7.1
BMI (kg/m^2^) (Mean+std deviation)		28.7±3.7
Incontinence Duration (year) (Mean+std deviation)		4.6±2.6
Operation Time (min) (Mean+std deviation)		35.7±2.1
Parity(Mean+std deviation)		3.4±1.3
Marriage Period		25.52±6.45
Number of children		3.12±0.92
Sexual intercourse frequency		1.02±0.57
Education Status (n,%)	High school and below	54 (75%)
	University and above	18(25%)
Education of spouse(n,%)	High school and below	31(43.15%)
	University and above	41(56.9%)
Prevention method(n,%)	Modern method	58 (80.6%)
	Retraction	14 (19.4%)
Type of Birth(n,%)	Normal Birth	49 (68.1%)
	Caesarean	23(31.9%)

No early period prolapse as surgery failure was observed in any patient. In none of the patients permanent urinary retention was observed. Only in seven cases, de novo urgency was observed which was successfully corrected with a short period of anticholinergic treatment. In addition, in nine patients, de novo dysuria was observed with no infection in urinary culture but regressed in a short period. In nine patients, forced and intermittent urination was observed with an approximately 100cc post voiding residual volume. In these patients 3-5 days intermittent catheterization was applied, and regression in complaints was observed. In one case, there was an intraoperative urethral injury which was repaired primarily by urologists. In 26 patients postoperative inguinal pain was observed which was treated well with anti-inflammatory and anti-analgesic drugs and physical exercise. In one patient, mesh exposure was observed and treatment was obtained with primary repair under local anesthesia and antibiotics. In 11 patients, postoperative dyspareunia was observed. All complications are shown in Table-2.

**Table 2:** Perioperative Complications due to Mesh Implantation.

Complications	Number*	Percent (%)
Permanent Urinary Retention	None	None
Transient Urinary Retention	9	12.5
*De novo* urgency	7	9.7
*De novo* dysuria	9	12.5
Bladder and Urethral Injury	1	1.3
Pelvic Pain	26	36.1
Mesh Erosion	None	None
Mesh Exposure	1	1.3
Major vessel and nerve Injury	None	None
Dyspareunia	11	15.2

* n:72

## Ratios

When the data were described as mean±standard deviation, there was a statistically significant increase in FSFI scores when compared preoperatively and postoperative 3^rd^ month, respectively as 21.2±1.9 and 24.4±1.8 (p <0.001). When the 1st-year FSFI scores were compared with preoperative scores, there was a statistically significant increase at postoperative first-year scores as well with the level of 27.9±1.4 (p <0.001). When the cut-off value for the FSFI score was 26.55, the rates of patients having sexual function disorder preoperatively, postoperative 3rd month and postoperative 1^st^ year were 100%, 86% and 16% respectively. According to these results, sexual function improved at the end of the first year. The results of the study are shown in Table-3.

**Table 3:** Preoperative, postoperative 3rd month and 1st year FSFI scores of patients undergoing mesh implantation.

FSFI Scores	Pre-operative	Post-operative (3th Month)	Post-operative (1th Year)
Mean±Std. Deviation	21.2±1.9	24.4±1.8	27.9±1.4
Increase in average %	–	15.1	
SFD Rate (%)	100	86	16
p-value	–	*<0.001	** <0.001

**SFD** = Sexual Function Disorder; P<0.05 was shown statistically;

* Pre-operative- Post-operative (3th Month);

** Pre-operative- Post-operative (1th Year)

## DISCUSSION

POP may lead to altered body image and may have adverse effects on desire, arousal, and orgasm. Furthermore, apart from urinary or prolapse symptoms, as women age, postmenopausal state and chronic diseases may have detrimental effects on sexual functions. It has been shown that older adults desire sexuality when there is a partner and they are in good health state (18). The POP and incontinence surgery improved FSFI scores and sexual functions in approximately 70% of patients, although some studies that rely on the use of non-condition-specific questionnaires as outcome measures indicate no change (19). We should note that there was an anatomically and functionally significant improvement in patients after the application of four-arm mesh implant.

POP also has a negative impact upon women's emotional health and subjective well-being. POP at a low grade can be successfully treated with conservative management including behavioral modification and pelvic floor muscle exercise. Nevertheless, increasingly POP patients are resorting to surgical intervention to improve their quality of life (20). In this study, the patients reported that the pre-operative anatomical condition caused emotional stress in them and they had gain self confidence after the operation.

POP treatment can be performed surgically or conservatively. There are still controversies among the best surgical technique, which includes; open surgery, laparoscopic or robotic abdominal sacrocolpopexy or anterior and posterior colporrhaphy with or without vaginal hysterectomy. The vaginal repair can be done with or without mesh. The use of mesh has increased due to functional and anatomic improvements when used in appropriate patients (10). In our study, we used four-armed non-absorbable, macroporous monofilament polypropylene synthetic mesh. With the vaginal mesh we have achieved the same functional results with fewer complications.

UI surgery or vaginal dissection may damage vascular and/or neuronal structures which may affect vaginal sense and orgasm (21). It should be kept in mind that although there is normal functioning anatomy, sexual function may be affected by other factors. In our study, we observed a positive effect of UI and POP surgery on the sexual lives of our patients at postoperative 1st year evaluation. Although this overall positive effect on sexual functions in our study shows similar results with other studies, some individual parameters such as sexual desire, orgasm, satisfaction show different results. In our study, there was a significant difference between postoperative 3rd month and 1st year results and we think this was due to patient's fear of organ damage with intercourse at an early time like three months. When we compared the postoperative 1st year results with preoperative period the significant change was due to sexual dysfunction that arises from UI and POP at the preoperative period. The functional and anatomic correction have an important effect on self-esteem which has a significant positive effect on sexual function disorder, even with a relative evaluation.

Results of the studies evaluating the effects of pelvic floor surgery on sexual functions show differences because of the demographic data differences between groups, differences in evaluation methods, stress factors in life, the relationship between partners, age, cultural characteristics and presence of menopause or chronic diseases. The fear of damaging organs after surgery may change the perception of genital health in both partners and may have a negative effect on sexual function. Because of all these reasons, it is hard to say that pelvic floor surgery improves sexual functions in all patients. It is important to provide consultancy about the postoperative sexual function improvement potential and the probable changes in sexual functions to patients who underwent UI and/or POP surgery.

## Limitation

The limitations of our study were a limited number of patients and the short period of post-operative observation.

## CONCLUSIONS

Transvaginal mesh use in the surgical treatment of pelvic organ prolapse improves quality of life. However, risk factors such as transvaginal mesh usage indication, surgical technique and experience of the surgeon, suitability of the material, the current health status of the patient and postoperative personal care of the patient may affect the success of operations. However, randomized controlled trials with more patients are needed.

## COMPLIANCE WITH ETHICAL STANDARDS

Adana City Teaching and Research Hospital Ethical Committee (Adana, Turkey) approved the study protocol. Written informed consent was obtained from all patients included in this study, and this study was conducted in agreement with the Declaration of Helsinki for Medical Research Involving Human Subjects.
